# The Dose Response Effects of Digital HIV Care Navigation on Mental Health and Viral Suppression Among Young People Living With HIV: Single-Arm, Prospective Study With a Pre-Post Design

**DOI:** 10.2196/33990

**Published:** 2022-07-18

**Authors:** Sean Arayasirikul, Caitlin M Turner, Dillon Trujillo, Jarett Maycott, Erin C Wilson

**Affiliations:** 1 Center for Public Health Research San Francisco Department of Public Health San Francisco, CA United States; 2 Department of Epidemiology and Biostatistics University of California, San Francisco San Francisco, CA United States; 3 Fielding School of Public Health University of California, Los Angeles Los Angeles, CA United States

**Keywords:** HIV, digital HIV care navigation, young people living with HIV, mHealth, mental health, viral suppression, health equity, digital health, equity, effect, suppression, disparity, men who have sex with men, MSM, transgender, sex

## Abstract

**Background:**

The HIV epidemic has revealed considerable disparities in health among sexual and gender minorities of color within the Unites States, disproportionately affecting cisgender men who have sex with men (MSM) and trans women. Social inequities further disadvantage those with intersectional identities through homophobia, antitrans discrimination, and racism, shaping not only those at risk for HIV infection but also HIV prevention and care outcomes. Digital interventions have great potential to address barriers and improve HIV care among cisgender MSM and trans women; however, efficacy of digital HIV care interventions vary and need further examination.

**Objective:**

This study assessed the 12-month efficacy of a 6-month digital HIV care navigation intervention among young people living with HIV in San Francisco, California. We examined dose-response relationships among intervention exposure (eg, text messaging), viral suppression, and mental health. Health electronic navigation (eNav) is a 6-month, text message–based, digital HIV care navigation intervention, in which young people living with HIV are connected to their own HIV care navigator through text messaging to improve engagement in HIV primary care.

**Methods:**

This study had a single-arm, prospective, pre-post design. Eligibility criteria for the study included the following: identifying as cisgender MSM or trans women, being between the ages of 18 and 34 years, being newly diagnosed with HIV, or not being engaged or retained in HIV care or having a detectable viral load. We assessed and analyzed sociodemographics, intervention exposure, and HIV care and mental health outcome data for participants who completed the 6-month Health eNav intervention. We assessed all outcomes using generalized estimating equations to account for within-subjects correlation, and marginal effects of texting engagement on all outcomes were calculated over the entire 12-month study period. Finally, we specified an interaction between texting engagement and time to evaluate the effects of texting engagement on outcomes.

**Results:**

Over the entire 12-month period, this study shows that every one-text increase in engagement was associated with an increased odds of undetectable viral load (adjusted odds ratio 1.01, 95% CI 1.00-1.02; *P*=.03). Mean negative mental health experiences decreased significantly at 12 months compared to baseline for every one-text increase in engagement (coefficient on interaction term 0.97, 95% CI 0.96-0.99; *P*<.01).

**Conclusions:**

Digital care navigation interventions including Health eNav may be a critical component in the health delivery service system as the digital safety net for those whose social vulnerability is exacerbated in times of crisis, disasters, or global pandemics owing to multiple social inequities. We found that increased engagement in a digital HIV care navigation intervention helped improve viral suppression and mental health—intersecting comorbid conditions—6 months after the intervention concluded. Digital care navigation may be a promising, effective, sustainable, and scalable intervention.

**International Registered Report Identifier (IRRID):**

RR2-10.2196/16406

## Introduction

### Background

The HIV epidemic has revealed considerable disparities in health among sexual and gender minorities of color within the Unites States. This is most evident among cisgender men who have sex with men (MSM) and who accounted for 69% of new HIV diagnoses in in 2019 [1]. Transgender—or trans—people are also a high-impact population despite representing 2% of new HIV diagnoses in 2019 [1]. Between 25% and 28% of trans people are HIV-positive [2]. Trans women are overwhelmingly impacted by HIV infection compared to other trans people, representing 93% of HIV diagnoses for trans adults and adolescents [1]. HIV infection impacts those at the intersection of minority race or ethnicity, sexual orientation, and gender identity contributing to grave health disparities among cisgender MSM and trans women. Black or African Americans represent nearly half of HIV prevalence estimates (46%) for trans women, and over one-third (38%) of HIV diagnoses among cisgender MSM [1].

There are more factors at play that contextualize the drastic disparities among trans women and cisgender MSM. For example, in a meta-analysis of HIV prevalence in the US transgender population estimates that 37% of trans women reported having engaged in sex work, 36% reported the use of an illicit substance, only 39.2% reported being employed, and 30.3% of trans women and trans men reported homelessness or unstable housing [2]. These social determinants of health are not only associated with a higher risk of HIV infection but combined with antitrans discrimination and racism, create barriers to care and are associated with suboptimal HIV care outcomes [3].

Digital interventions have great potential to address barriers and improve HIV care among cisgender MSM and trans women. A systematic review of digital interventions found that overall, digital interventions had a positive impact on HIV care outcomes [4]. A Cochrane review found that interventions using mobile phone text messaging at weekly intervals was effective in increasing adherence to HIV treatment or antiretroviral therapy (ART) [5]. Another study with a sample of trans women living with HIV in Los Angeles, California, tested an automated, unidirectional, text messaging intervention and found that it resulted in a number of HIV care outcome improvements including retention to care and ART adherence [6]. Efficacy can vary among digital intervention components and across HIV outcomes with the most successful interventions combining approaches [4]. Finitis et al [7] conducted a systematic review of text messaging interventions among people living with HIV and found that interventions that supported bidirectional communication, occurred less frequently than daily, included personalized message context, and corresponded with participants’ ART dosing schedule led to larger effects on ART adherence compared to standard of care controls. This study examines the impact of a multicomponent digital HIV care navigation intervention on mental health and viral suppression among young people living with HIV in San Francisco, California.

### Overview of Health eNavigation

Health eNavigation (eNav) is a 6-month text message–based, digital HIV care navigation intervention where young people living with HIV were connected to their own digital HIV care navigator through bidirectional text messaging to improve engagement in HIV primary care. The intervention included delivery of personalized messages and content that addressed the following topics: (1) HIV care navigation, (2) health promotion and education, (3) motivational interviewing (MI), and (4) social support. HIV care navigation guides participants in knowing where, when, and how to access all health and related services, and increases access to appropriate resources (eg, primary medical care, mental health care, housing, insurance and benefits, etc) [8]. Health promotion and education ensures optimal health literacy for all participants by providing information on the biology of HIV, disease management, communication with providers, risk reduction and healthy behavior, and medication adherence. MI is a technique and a style of counseling that can help resolve the ambivalence that prevents patients from realizing their personal goals [9,10]. Social support is provided through establishing an open, nonjudgmental care relationship between participants and their HIV care navigator to be patient-centered and address topics most important to young people living with HIV. Intervention components are described in depth in a prior study [11].

Informed by 2 health services frameworks, Health eNav transforms how HIV care navigation is delivered, seeking to improve health outcomes by amplifying the reach and value of the patient-centered medical home model and the chronic care model with the use of digital technology [12,13]. Health eNav provides participants with personalized engagement to strengthen the provider-patient relationship to eliminate barriers to care, and increase the efficiency and quality of care [12]. Health eNav delivers digital HIV care navigation by providing increased linkages to community resources in a community-driven, cost-effective way, promoting self-management that empowers participants to take an active role in their health, and offering clinical decision support, information sharing, and proactive care in real time [13].

## Methods

### Ethics Approval

All procedures performed in studies involving human participants were in accordance with the ethical standards of the institutional and national research committee and with the 1964 Helsinki declaration and its later amendments or comparable ethical standards. This study protocol was approved by the institutional review board at the University of California, San Francisco (IRB #16-19675).

### Study Design, Recruitment, and Data Collection

Data for this analysis were obtained from the Health eNav study at San Francisco Department of Public Health (2017-2018). Health eNav was a digital care navigation intervention designed to improve HIV care linkage and retention and subsequent viral suppression among young cisgender MSM and trans women living with HIV. A digital care navigator delivered the intervention via bidirectional text messaging. This is a single-arm, prospective, pre-post design study. Study procedures are described in depth in a prior study [11].

Eligibility criteria for the study included the following: identifying as cisgender MSM or trans women, being between the ages of 18 and 34 years, and being newly diagnosed with HIV or not being engaged or retained in HIV care or having a detectable viral load. Participants were recruited via convenience sampling from 5 clinics and community-based organizations in San Francisco serving young people living with HIV. If eligible, participants met with research staff at study offices within the San Francisco Department of Public Health, where informed consent was obtained. Of 170 individuals screened, 140 were eligible. However, 20 were subsequently lost to follow-up and were not enrolled. This left a final sample of 120 young cisgender MSM or trans women living with HIV.

This analysis examines data collected from computer-assisted self-interview surveys of self-report data administered at baseline, 6 months, and 12 months and intervention exposure data that characterize the number of text messages sent during the 6-month intervention period. Intervention exposure data were collected and exported on the backend of our text messaging platform.

### Measures

#### Demographics

We analyzed the following sociodemographic information: age at interview (in years), gender identity (trans woman vs man), race or ethnicity (non-Hispanic, Latinx American Indian, or Alaska Native; Asian; Black or African American; Multiracial; White; or Hispanic or Latinx), education level (high school or General Educational Development or at least a college education), current living situation (stable vs unstable), income level in the last month (US $0-250, US $251-600, US $601-1300, or ≥US $1301), and incarceration status in the last 6 months.

#### HIV Care Continuum Outcomes

We assessed 3 key HIV care continuum outcomes at baseline, 6 months, and 12 months of follow-up: whether participants received primary HIV care within the 6 months prior to their study visit, whether participants were currently taking HIV medications at each of these study visits, and whether participants had an undetectable viral load at each of these study visits.

#### Mental Health

We measured mental health using the mental health subscale of the 12-item Short-Form Health Survey [14]. A composite score was generated by summing responses to items ranging from 0=“none of the time” to 5=“all of the time” and detailing the frequencies of the following experiences in the last month: feeling calm and peaceful, having a lot of energy, feeling downhearted and blue; and physical health or emotional problems interfering with social activities. The first 2 items in this list were reverse-scored before creating the composite variable.

#### HIV-Related Stigma

To measure HIV stigma, we used the shortened revised HIV stigma scale tailored for young people living with HIV [15]. A composite score was generated from summing responses (from 0=“Strongly disagree” to 4=“Strongly agree”) to items such as: loss of friends after disclosing HIV status, feeling like a bad person owing to HIV status, or feeling like most people with HIV are rejected when others find out. For both the mental health and stigma composite scores, higher scores denoted a higher number of mental health issues and stigma experiences, respectively.

#### Intervention Exposure

We measured the number of text messages sent between participants and the digital navigator during the 6-month intervention period, summing the number of text messages. From this, we created the intervention exposure variable, “texting engagement level,” defined as the total number of texts sent or received by each participant over the 6-month period. Text message conversations did not comprise preprogrammed, automated, repeated texts; instead, text messages were bidirectional and delivered by an interventionist in conversation with participants using motivational interviewing techniques to have conversations personalized to participants’ individual needs. For example, if a participant identified a need for social support to cope with a new HIV diagnosis, the conversation would center that topic. Alternatively, if a participant needed information about health insurance or a health care appointment or medication adherence reminder, the digital HIV care navigator would provide that information or provide follow-up tailored to participants’ individual needs. To ensure a baseline level of engagement in the case that participants were not initiating text messages, the digital navigator attempted to start a conversation with participants by sending one text message each week over the duration of the intervention period. The number of text messages ranged from 24 to 467 text messages.

### Statistical Analysis

Of the 120 participants in Health eNav, we restricted our analysis to the 60 participants who completed the 6-month digital care intervention. Participants who did not complete the intervention included people who moved out of our jurisdiction, were incarcerated, withdrew from the study, or lost to follow-up during the intervention period. We hypothesized that participants who completed the 6-month digital HIV care navigation intervention represent a different intervention and outcome experience from those who did not. As a result, analyses were restricted to intervention completers. Additionally, we excluded 4 participants who experienced interruptions in their phone service, lost their phone for a period of time, or deleted the text messaging app, and as a result, text messaging was not possible. The final analytic sample comprised 56 participants.

First, we characterized the entire sample with baseline sociodemographic data. We then described the mean texting engagement level by sociodemographics, HIV care continuum outcomes, mental health composite score (dichotomized into “low mental health issues” or a score of 0 to 10 vs “high mental health issues” or a score of 11 to 20), and HIV-related stigma composite score (dichotomized into “low HIV-related stigma experiences” or a score of 0 to 15 vs “high HIV-related stigma experiences” or a score of 16 to 30). Given the hypothesized difference in intervention effects from baseline to 6 months and then to 12 months, we assessed all outcomes (HIV care continuum, mental health, and HIV-related stigma outcomes) for a 6-month intervention period and 12-month intervention period using generalized estimating equations (GEE) to account for within-subjects correlation. Marginal effects of intervention exposure (or texting engagement) on all outcomes were calculated over the entire 12-month study period using GEE models. Finally, following the logic of differential intervention effects at 6 and 12 months, we specified an interaction between intervention exposure and time point to evaluate the possible effects of a dose response on all 5 outcomes by 6 months and 12 months. All statistical analyses were conducted in Stata 14 [16]. Comparisons producing *P* values less than .05 were considered statistically significant.

## Results

Table 1 displays the distribution of baseline sociodemographics, HIV care continuum outcomes, mental health issues, and HIV-related stigma experiences with accompanying mean intervention exposure (or texting engagement). Younger participants, trans women, Hispanic or Latinx participants and those with multiple races or ethnicities, those with less than a college education, those with unstable housing, those with higher incomes, and those who were recently incarcerated had higher mean texting engagement. Those who did not recently receive HIV care, were not currently taking ART, and those with an undetectable viral load also had higher mean texting engagement. Finally, we observed higher mean texting engagement among those who had higher mental health and HIV-related stigma composite scores.

Table 2 shows that the odds of undetectable viral load increased over the initial 6-month intervention period (odds ratio [OR] 2.07, 95% CI 1.04-4.11; *P*<.01) and the 12-month period (OR 2.98, 95% CI 1.11-8.04; *P*=.03). Mean negative mental health decreased over the 6-month period (mean change estimate 0.18, 95% CI 0.05-0.58; *P*<.01), but not over the 12-month period. All other outcome models produced nonsignificant results.

GEE models over the entire 12-month study period (Table 3) showed that every one-text message increase in engagement or intervention exposure was associated with an increased odds of undetectable viral load (adjusted OR 1.01, 95% CI 1.00-1.02; *P*=.03) and a mean increase in HIV-related stigma experiences (mean change estimate 1.03, 95% CI 1.01-1.05; *P*=.02). To better understand the impact on HIV-related stigma, we conducted a post hoc analysis where we stratified by the timing of HIV diagnosis (within the last year vs prior to the last year) given that the recency of HIV diagnosis could serve as a critical period in which young people living with HIV are particularly vulnerable to HIV stigma. Text messaging was associated with a mean increase in HIV stigma only among those who were recently diagnosed (estimate 1.04, 95% CI 1.01-1.07; *P*<.01). Finally, in testing the effect of intervention engagement on outcomes for the 6-month period compared to the 12-month period, we found that mean negative mental health experiences decreased significantly at 12 months compared to baseline for every one-text increase in engagement (coefficient on interaction term 0.97, 95% CI 0.96-0.99; *P*<.01).

**Table 1:** Sociodemographics, HIV care continuum outcomes, mental health, and HIV-related stigma among young cisgender men who have sex with men and trans women living with HIV who completed the intervention, overall and by texting engagement, Health eNavigation (N=56; 2017-2019).

Sociodemographics				Baseline, n (%)^a^		Texting engagement level, mean (SD)
**Age (years)**						
	18-24			10 (17.86)		141.60 (55.55)
	25-36			46 (82.14)		138.65 (66.26)
**Gender identity**						
	Trans woman			8 (14.29)		146.75 (48.21)
	Cisgender Man			48 (85.71)		137.92 (66.62)
**Race or ethnicity**						
	Black, non-Hispanic or Latinx			11 (19.64)		139.82 (61.57)
	Hispanic or Latinx			14 (25.00)		156.57 (62.65)
	Multiple races, non-Hispanic or Latinx			14 (25.00)		155.14 (73.83)
	White, non-Hispanic or Latinx			17 (30.36)		111.29 (52.77)
**Education level**						
	High school or General Educational Development or less			21 (37.50)		149.05 (69.21)
	Some college or more			35 (62.50)		133.26 (54.42)
**Current living situation**						
	Unstable			35 (62.50)		145.57 (61.31)
	Stable			21 (37.50)		128.52 (68.47)
**Income in the last month (US $)**						
	601-1300			13 (23.21)		152.15 (66.45)
	251-600			16 (28.57)		125.56 (64.85)
	0-250			14 (25.00)		133.79 (53.17)
	≥1301			13 (23.21)		148.77 (74.04)
**Incarcerated, last 6 months**						
	Yes			7 (12.50)		146.57 (55.81)
	No			49 (87.50)		138.12 (65.55)
**HIV care continuum outcomes**						
	**Received primary HIV care, last 6 months**					
		Yes	50 (89.29)		136.10 (64.01)	
		No	6 (10.71)		164.83 (59.88)	
	**Currently taking antiretroviral therapy**					
		Yes	47 (83.93)		135.66 (63.75)	
		No	8 (14.29)		145.38 (58.73)	
	**Undetectable viral load**					
		Yes	36 (64.29)		146.33 (63.12)	
		No	16 (28.57)		109.69 (53.36)	
**Mental health and HIV-related stigma outcomes**						
	**Mental health composite score (0-20)**					
		High mental health issues (11-20)	23 (41.07)		149.17 (72.72)	
		Low mental health issues (0-10)	33 (58.93)		132.21 (57.32)	
	**HIV-related stigma composite score (0-30)**					
		High stigma experiences (16-30)	17 (30.36)		171.71 (67.07)	
		Low stigma experiences (0-15)	39 (69.64)		125.00 (57.94)	

^a^Percentages calculated out of total number of participants at baseline who completed the intervention and were included in the analysis (N=56), unless otherwise specified.

**Table 2:** Differences in HIV care continuum, mental health, and HIV-related stigma outcomes at baseline and 6 months for cisgender men who have sex with men and trans women living with HIV who completed the intervention, Health eNavigation (2017-2019).

				Outcomes of generalized estimating equations^a^ over time: 6 months compared to baseline					Outcomes of generalized estimating equations^a^ over time: 12 months compared to baseline		
				Effect estimate^b^ (95% CI)		*P* value		Effect estimate^b^ (95% CI)			*P* value
**HIV care continuum outcomes**											
	**Received primary HIV care, last 6 months**										
		No	Reference					Reference			
		Yes	3.11 (0.56-17.18)		.19		0.67 (0.21-2.10)			.49	
	**Currently taking antiretroviral therapy**										
		No	Reference					Reference			
		Yes	0.75 (0.35-1.61)		.46		1.38 (0.42-4.55)			.59	
	**Undetectable viral load**										
		No	Reference					Reference			
		Yes	2.07 (1.04-4.11)		.04		2.98 (1.11-8.04)			.03	
**Mental health and HIV-related stigma outcomes**											
	Mental health composite score			0.18 (0.05-0.58)		<.01		0.41 (0.14-1.24)			.12
	HIV-related stigma composite score			0.29 (0.05-1.75)		.18		0.21 (0.03-1.22)			.08

^a^Five models were created using generalized estimating equations to estimate the effects of each outcome over a 6- and 12-month intervention period. These models produced odds ratios for dichotomous outcomes and prevalence ratios for continuous outcomes.

^b^Odds ratios for dichotomous outcomes; mean change for continuous outcomes.

**Table 3:** Differences in HIV care continuum, mental health, and HIV stigma outcomes over 12 months by mean texting engagement for cisgender men who have sex with men and trans women living with HIV who completed the intervention, Health eNavigation (2017-2019)^a^.

				GEE effects texting engagement over the 12-month study period		
				Adjusted effect estimate^b^ (95% CI)		*P* value
**HIV care continuum outcomes**						
	**Received primary HIV care, last 6 months**					
		No	Reference			
		Yes	1.00 (0.99-1.00)		.29	
	**Currently taking ART**					
		No	Reference			
		Yes	1.00 (0.99-1.01)		.75	
	**Undetectable viral load**					
		No	Reference			
		Yes	1.01 (1.00-1.02)		.03	
**Mental health and HIV-related stigma outcomes**						
	Mental health composite score		1.00 (0.99-1.02)		.61	
	HIV-related stigma composite score		1.03 (1.01-1.05)		.02	

^a^Five models were created using generalized estimating equations to estimate the effects of each outcome over the entire 12-month period. These models produced odds ratios for dichotomous outcomes and prevalence ratios for continuous outcomes.

^b^Odds ratios for dichotomous outcomes; mean change for continuous outcomes.

## Discussion

### Principal Findings

Our study found evidence of dose-response effects associated with increases in intervention exposure or text message engagement that led to improved odds of undetectable viral load and decreases in negative mental health experiences. While there are studies supporting the application of mobile health (mHealth) approaches to improve HIV care continuum outcomes such as viral suppression, similar advances at the intersection of mental health and HIV care have lagged [17]. A recent study found that HIV medication adherence was negatively associated with poor mental health experiences such as depression, trauma, and adverse childhood experiences [18]. Saberi et al [18] conducted in-depth interviews with 29 participants and found that young people living with HIV preferred digital approaches to mental health service delivery. Our findings strengthen the use of text messaging interventions to support both mental health and HIV care.

We also found that as text messaging increased, HIV stigma experiences also increased. The mean increase in HIV-related stigma experiences associated with increased engagement in text messaging was a surprising finding. Our post hoc analysis found that text messaging was associated with a mean increase in HIV-related stigma only among those who were recently diagnosed (estimate 1.04, 95% CI 1.01-1.07; *P*<.01), suggesting that HIV diagnosis timing might drive the relationship between texting and HIV-related stigma over the study period. We suspect that people who were recently diagnosed either needed more support to process their diagnosis or the changes in their identity related to their diagnosis; participating in an intervention to improve their engagement in HIV care may have brought HIV-related stigma experiences to the surface in order to be addressed as a potential barrier to HIV care engagement in conversations with their digital HIV care navigator [19]. Though studies have called for differentiated service delivery models for key populations experiencing multiple forms of stigma related to HIV and intersectional identities [20], few mHealth interventions have examined the unique needs of young people recently diagnosed with HIV [19].

### Limitations and Future Research

Our results should be interpreted with a number of limitations in mind. First, results from our sample of young cisgender MSM and trans women living with HIV in San Francisco may not generalize to other populations. Similarly, since we included only those who completed the 6-month digital care navigation intervention, the findings may only apply to young cisgender MSM and trans women living with HIV who adhere to interventions of this nature. This intervention was focused on changing how HIV care navigation was implemented to include digital methods for young people living with HIV, and owing to our local epidemic at the time of enrollment, this included both cisgender MSM and trans women. We hypothesized that both groups would benefit from participating because the digital navigation participants received was tailored to their individual needs. We did not sample participants to detect differences between these 2 groups. Measurement bias may be in issue as well. Texting engagement, defined as number of texts sent during the digital care navigation component of the intervention, precludes depictions of texting patterns on a day-by-day basis. Texting engagement could have been intermittent as well. However, restricting to those who completed this component of the intervention insured that texting patterns were likely consistent over the study period. While this intervention did not use standardized, preprogrammed text messages, our training and approach using MI as a client-centered communication framework was standardized and focused on supporting change talk. Selection bias may have played a role in our study as well. Participants who were actively engaged in substance use or encountering acute housing instability may not have had the time or capacity to participate in our intervention study. Finally, given the small sample size, it is possible that some of our analyses were underpowered to detect true effects.

### Implications for Future Studies and Conclusions

The COVID-19 pandemic has disrupted the status quo systems of HIV care [21-23], renewing the critical importance of digital interventions in a time of intersecting epidemics [24]. Digital care navigation interventions including Health eNavigation may be a critical component in the health delivery service system as the digital safety net for those whose social vulnerability is exacerbated in times of crisis, disasters, or global pandemics owing to multiple social inequities [25]. We found that increased engagement in a digital HIV care navigation intervention helped improve viral suppression and mental health—intersecting comorbid conditions—6 months after the intervention ended. Digital care navigation may be a promising, sustainable, and scalable intervention for not only making personalized health care more accessible [26], but also serve as a critical link in centering the whole person in a learning health system [27-29].

## Abbreviations

ART: antiretroviral therapy
eNav: electronic navigation
GEE: generalized estimating equation
mHealth: mobile health
MI: motivational interviewing
MSM: men who have sex with men
OR: odds ratio
